# Contactless photomagnetoelectric investigations of 2D semiconductors

**DOI:** 10.3762/bjnano.9.256

**Published:** 2018-10-25

**Authors:** Marian Nowak, Marcin Jesionek, Barbara Solecka, Piotr Szperlich, Piotr Duka, Anna Starczewska

**Affiliations:** 1 Institute of Physics, Center for Science and Education, Silesian University of Technology, Krasińskiego 8, 40-019 Katowice, Poland

**Keywords:** carrier mobility, contactless investigations, graphene, photomagnetoelectric effect, 2D materials

## Abstract

**Background:** Applications of two-dimensional (2D) materials in electronic devices require the development of appropriate measuring methods for determining their typical semiconductor parameters, i.e., mobility and carrier lifetime. Among these methods, contactless techniques and mobility extraction methods based on field-effect measurements are of great importance.

**Results:** Here we show a contactless method for determining these parameters in 2D semiconductors that is based on the photomagnetoelectric (PME) effect (also known as the photoelectromagnetic effect). We present calculated dependences of the PME magnetic moment, evoked in 2D Corbino configuration, on the magnetic field as well as on the intensity and spatial distribution of illumination. The theoretical predictions agree with the results of the contactless investigations performed on non-suspended single-layer graphene. We use the contactless PME method for determining the dependence of carrier mobility on the concentration of electrons and holes induced by a back-gate voltage.

**Conclusion:** The presented contactless PME method, used in Corbino geometry, is complementary to the mobility extraction methods based on field-effect measurements. It can be used for determining the mobility and diffusion length of carriers in different 2D materials.

## Introduction

The application of two-dimensional (2D) materials in electronic devices [1–6] requires the development of appropriate measurement methods for determining their typical semiconductor parameters, i.e., carrier mobility (μ) and lifetime (τ). Among these methods, contactless techniques [7–8] and mobility extraction methods based on field-effect measurements [9] are of great importance. Here we show a contactless method for determining these parameters in 2D semiconductors that is based on the photomagnetoelectric (PME) effect [10].

There are a few phenomena which are or may be called PME effects. For example, in semiconductors the simultaneous action of light and magnetic field evokes specific electromotive forces [10]. In the magnetic field, *B*, perpendicular to the photogenerated carrier concentration gradient, the diffusing electrons and holes are deflected in opposite directions. Their current flows in a third, mutually perpendicular direction. The PME response decreases with the increase of recombination rate. Of course, the PME signal is stronger for higher μ. Therefore, investigations of the PME effect are used to determine carrier recombination and transport [10–14] as well as parameters describing the interaction of light with the investigated materials [15–17]. The PME effect is generally accepted for infrared photon detectors [18–20] which are based upon the proportionality of this effect on the intensity of radiation that enters the semiconductor.

**Figure 1:**
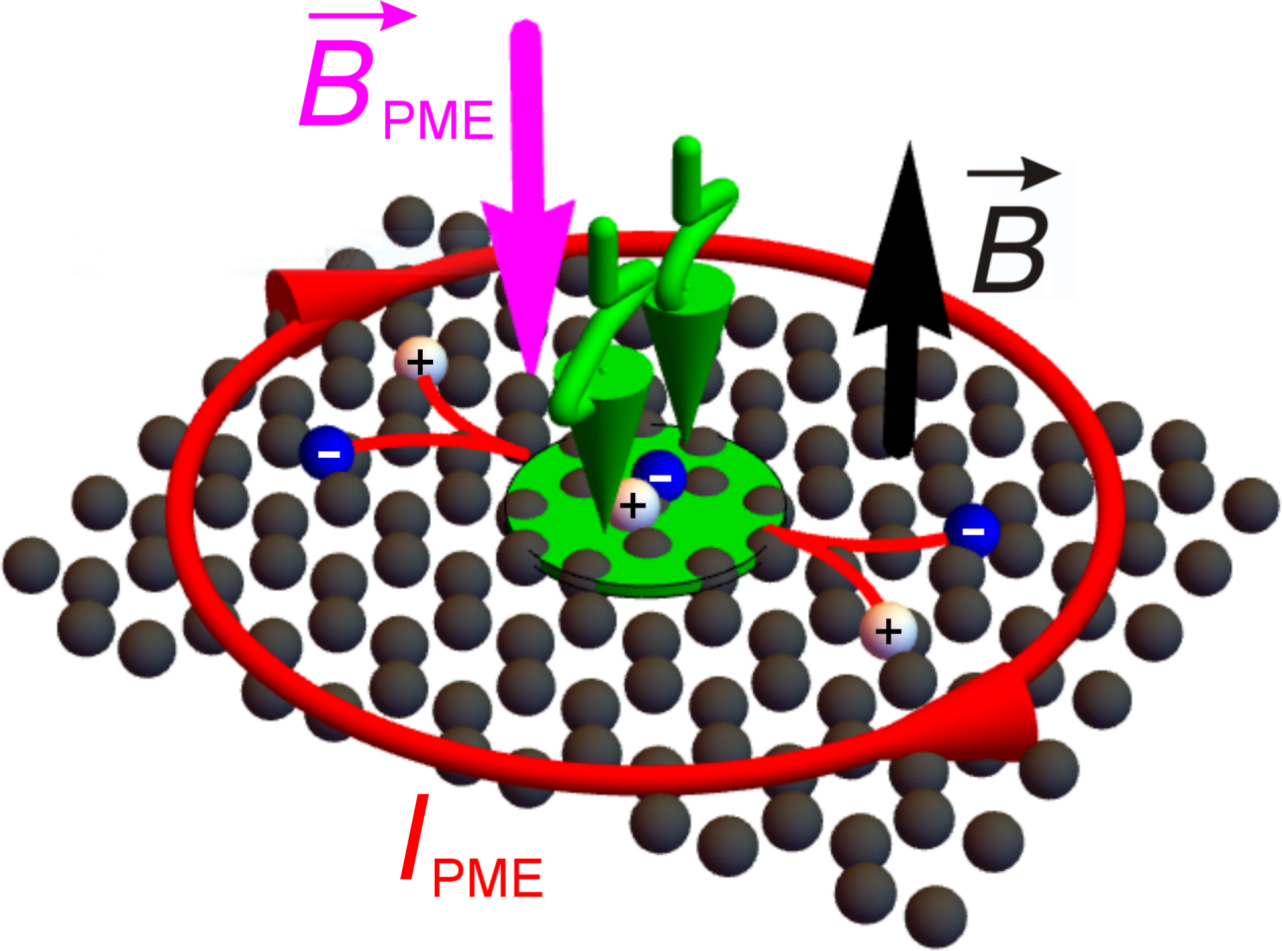
A schematic of the magnetic moment 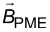 evoked by the PME circulating current in a point-illuminated 2D semiconductor in Corbino configuration (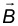
– vector of external magnetic field induction; the green wavey arrow represents illumination; the green, partially transparent circle shows the illuminated area of the sample; the plus and minus signs represent the photogenerated electrons and holes separated by the magnetic field; *I*_PME_ – the circulating PME current).

Mette [21] proposed a noncontact measurement involving the PME effect in Corbino configuration, i.e., in point-illuminated bulk semiconductors in Faraday geometry (Figure 1). In a sample illuminated by a circular spot of light, excess carriers generated by photons of appropriate energy diffuse in all directions. If this happens in a magnetic field perpendicular to the sample surface, diffusing carriers are deflected because of the Lorentz force. In this case, they flow around the illuminated region (Figure 1). The circulating PME-Corbino current evokes the PME magnetic moment. In the induction method of measurements, the varying PME magnetic moment caused by intermittent illumination induces an AC voltage in appropriate measuring coils.

The formula describing the PME magnetic moment, evoked in a bulk semiconductor wafer illuminated by weakly absorbed radiation as a function of τ, sample thickness, and surface recombination velocity (*s*) of the illuminated (front) and unilluminated (back) surfaces were previously developed in [22]. These theoretical predictions were in good qualitative agreement with results of spectral investigations on bulk Ge samples of varying τ and *s* [22–23]. However, it is not easy to deduce the proportionality coefficient between the voltage response (induced in measuring coil) and the investigated PME magnetic moment. A reliable determination of τ or *s* was possible only after calibrating the apparatus with standard samples of known semiconductor parameter values [22–23]. To avoid this disadvantage, Loncierz et al. [24] proposed the use of the relative dependence of PME response on the frequency of light-intensity modulation. This method was used to determine the carrier lifetimes in bulk samples of Si:B, GaAs:Te and GaAs:Si [24–26]. Observations of the PME effect in graphene were reported only in [27–28].

It should be noted that the Corbino geometry has a distinct advantage over the Hall and van der Pauw geometries, in that it provides a direct probe of the bulk two-dimensional electron gas without having complications due to edge state transport [29–30]. For example, the fractional quantum Hall ordering was investigated in two-terminal suspended graphene Corbino devices [31].

The purpose of the present work is to analyze the dependence of the integral magnetic PME moment in a 2D semiconductor on the mobility and lifetime of carriers. The influence of experimental conditions (magnetic field, frequency of illumination chopping, illumination intensity, and beam radius) is taken into consideration. To test the theoretical predictions, we apply the induction technique in PME investigations of single-layer graphene on polyethylene terephthalate (PET) foil. The main aim of our work is to use the PME method for determining the dependence of μ on the concentration of electrons and holes induced by a back-gate voltage in graphene.

## Theoretical Description

In the presented investigations, it was assumed that the 2D material is illuminated by a circular spot of TEM_00_ light with photon energy greater than its optical energy gap. The transport of electrons and holes through 2D samples in the presence of a steady magnetic field under photogeneration and recombination is described by the solution of Maxwell's equations, the Poisson equation, the continuity equations and the transport equations (see Supporting Information File 1 for more details). The circulating PME-Corbino current (Figure 1) causes the PME magnetic moment

[1]
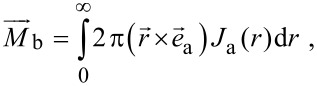


where 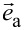 represents a vector of the azimuthal axis, *J*_a_(*r*) is the sum of azimuthal components of electron and hole current densities, 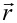 is the position vector, and *r* is the distance from the center of the light spot.

Assuming an absorption coefficient of light appropriate for graphene (α = 1/137 [32]), the theoretical formula presented in Supporting Information File 1, and using Wolfram Mathematica 10.0, we numerically calculated 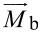 as a function of parameters describing the experiment: magnetic field (*B*), beam radius (*R*_b_) and maximum intensity (*I*_V0_) of the radiation incident upon a sample. These calculations were done for different values of τ and μ.

**Figure 2:**
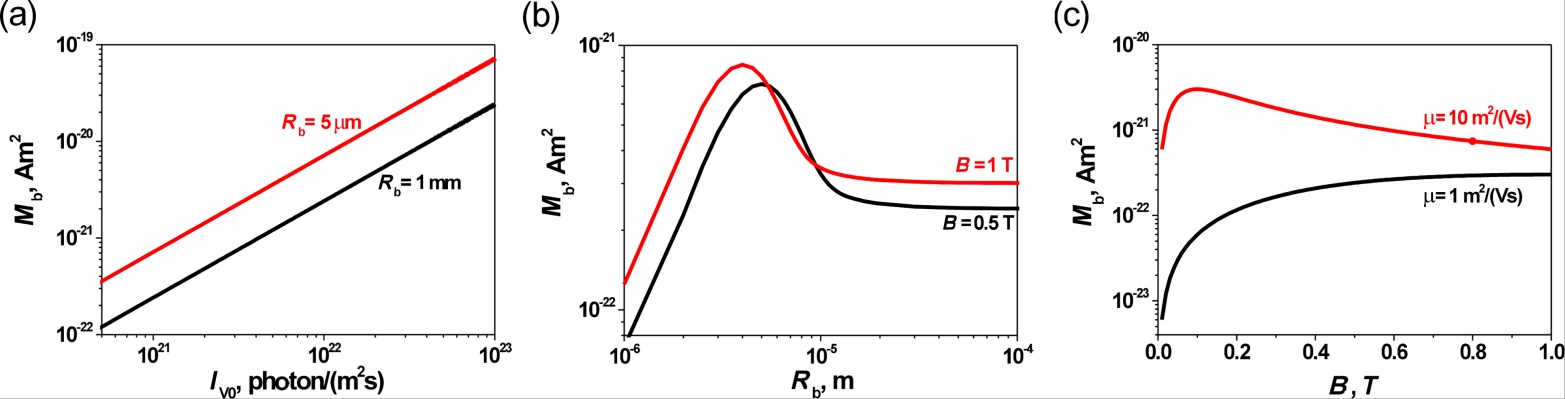
The calculated dependences of the PME magnetic flux: (a) 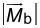 vs the illumination intensity for different values of the beam radius (*B* = 0.5 T; μ = 1 m^2^V^−1^s^−1^; τ = 10^−10^ s); (b) 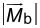 vs the beam radius for varying values of the magnetic field (μ = 1 m^2^V^−1^s^−1^; τ = 10^−10^ s; I_V0_ = 10^21^ photons/(m^2^s)); (c) 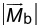 vs the magnetic field for varying values of carrier mobility (*R*_b_ = 1 mm; τ = 10^−10^ s; I_V0_ = 10^21^ photons/(m^2^s)).

Figure 2a presents the linear dependence of the PME magnetic flux (*M*_b_ = 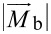) on the illumination intensity for different values of the beam radius. The *M*_b_ is a nonlinear function of *R*_b_ (Figure 2b) and *B* (Figure 2c). The *M*_b_ increases with increasing *R*_b_, reaches the maximum, and decreases to a constant value (Figure 2b). For a large value of μ, the *M*_b_ increases with the increase of *B*, reaches a maximum value, and then decreases (Figure 2c). For a small value of μ, the maximum of *M*_b_ is shifted to stronger magnetic fields (Figure 2c).

**Figure 3:**
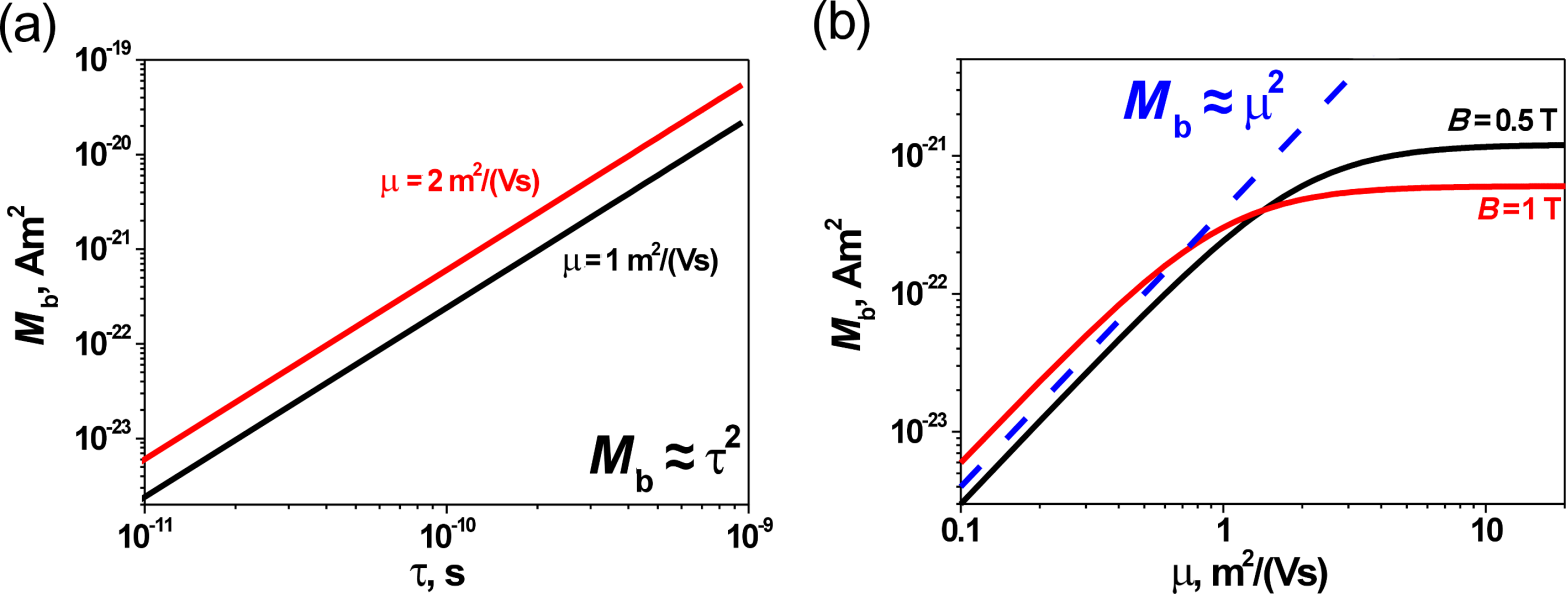
Theoretical dependence of the PME magnetic flux: (a) 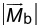 vs the carrier lifetime for different values of carrier mobility (*B* = 0.5 T; *R*_b_ = 1 mm; *I*_V0_ = 10^21^ photons/(m^2^s)); (b) 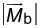 vs the carrier mobility at varying values of magnetic field (τ = 10^−10^ s, *R*_b_ = 1 mm, and *I*_V0_ = 10^21^ photons/(m^2^s)).

Figure 3 shows the calculated dependences of the PME magnetic flux on values of material parameters. The 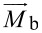 is proportional to the square of the carrier lifetime (Figure 3a). Its value increases with increasing μ and reaches its maximum value (Figure 3b). It should be noted that for a stronger magnetic field this final value of 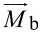
is reduced (Figure 3b). It is important that for a relatively weak magnetic field (*B* < 1 T) and for μ smaller than about 1 m^2^V^−1^s^−1^, the 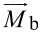 is proportional to the square of the carrier mobility (Figure 3b). Assuming the so-called Einstein relations of the proportionality between the carrier diffusion constant and μ, the diffusion length of carriers is proportional to the square root of carrier mobility and lifetime: 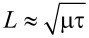. Hence, in the case of the small μ and weak magnetic field, the PME magnetic flux is proportional to the fourth power of the diffusion length of carriers (
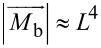
).

## Experimental

The investigated graphene samples were supplied by Graphene Supermarket. The single-layer graphene films were grown by CVD processing on a cooper foil and transferred onto a 170 μm thick polyethylene terephthalate (PET) foil. The polycrystalline graphene films covered about 90% of the foil, with occasional holes and cracks. The presence of single-layer graphene was confirmed by Raman spectroscopy using an NTEGRA Spectra (NT-NDT) device with a wavelength of 532 nm. The carrier mobility μ_e_ = 1256(25) cm^2^V^−1^s^−1^ and sheet carrier concentration *n*_e_ = 4.65(6)·10^16^ m^−2^ in the graphene were determined using the Van der Pauw method. For these measurements of resistivity and the Hall coefficient, the samples were equipped with 150 nm thick Au electrodes deposited using a Q150R ES rotary-pumped sputter coater with a film thickness monitor. For the PME-Corbino investigations of graphene with electrostatically tunable carrier density, a 150 nm thick Au film was also deposited on the back surface of the PET foil.

**Figure 4:**
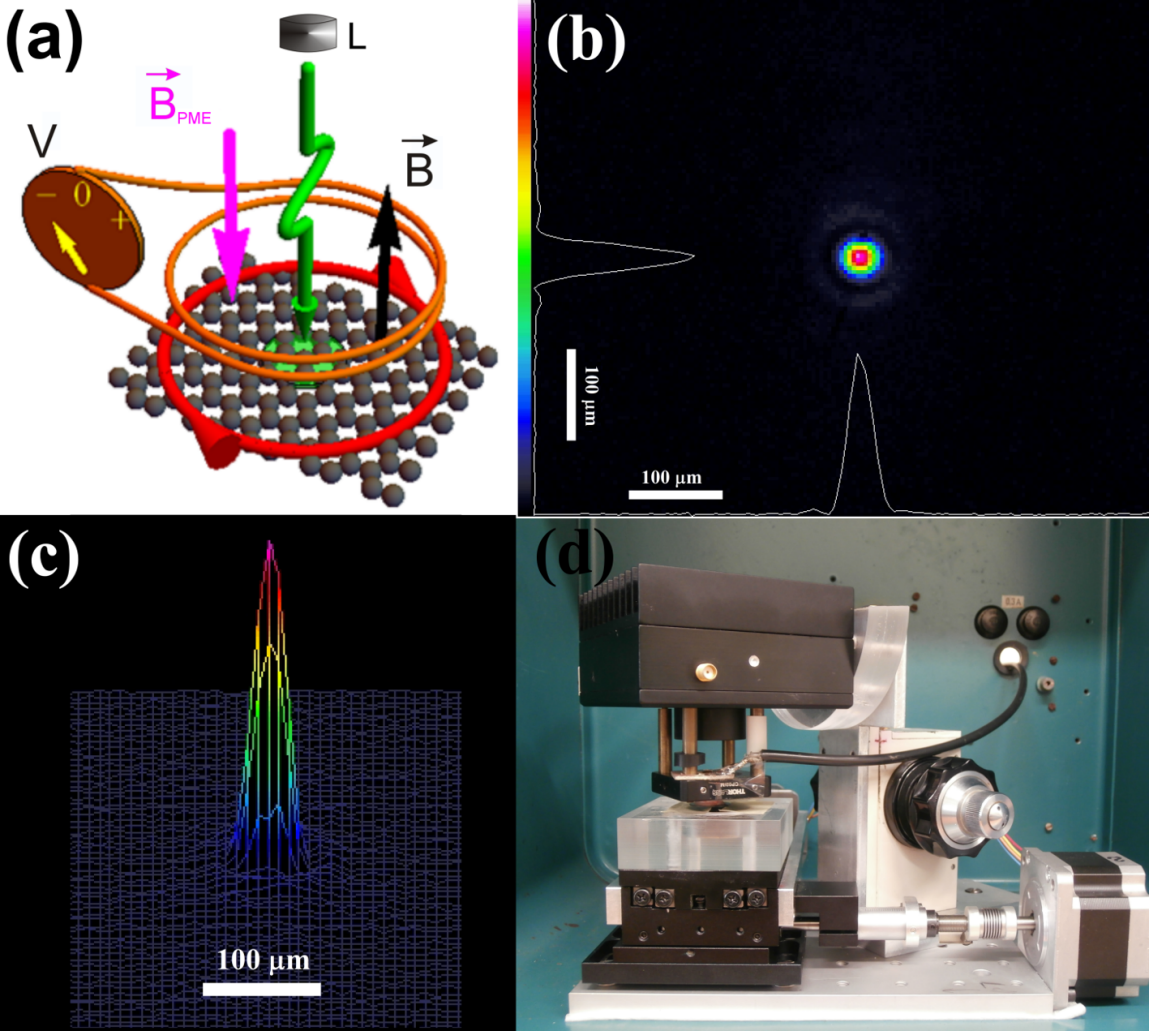
Schematic presentation (a) of the measuring set up used for the induction technique of PME investigations of a point-illuminated 2D semiconductor in Corbino configuration (V – nanovoltmeter connected to a measuring coil; L – electronically chopped light source; the other symbols have the same meanings as in Figure 1); (b) 2D and (c) 3D mapping of the illumination intensity; and (d) a picture of the main part of the measurement set up.

The current–voltage characteristics were recorded using a Keithley 6221 DC current source, Keithley 196 digital multimeter, and a Keithley 705 scanner. The measurements were carried out in magnetic fields from −0.725 T to 0.725 T using a DC electromagnet. The PME investigations were performed using the induction technique (Figure 4a). Under amplitude-modulated illumination of the sample, the PME circulating current varied. Consequently, the changing PME magnetic moment, caused by this alternating current, induced a measurable voltage in the suitably placed coil. In our experiments, the samples were placed in a slit between neodymium magnets and a measuring coil. The graphene was illuminated through a 2 mm diameter hole in one of the magnets using a temperature-stabilized laser diode, Sanyo DL7032-001 (λ = 830 nm, *P* = 100 mW). The laser diodes were biased with an ITC510 (Thorlabs) laser diode controller. In our experiments, the laser diode bias was sinusoidally modulated around a DC bias chosen to ensure that the waveform of the light output signal is sinusoidal with negligible distortion. The radiation intensity was changed using neutral density filters (UV-NIR-FILTER-250-2000 nm, quartz glass substrate, Oriel). The intensity was monitored using a Hamamatsu S3399 photodiode whose short-circuit current was measured using an EG&G 5110 lock-in amplifier. The time dependence of the illumination was determined using a Rigol Technologies DS1202CA oscilloscope. The spatial distribution of illumination (Figure 4b,c) was controlled using a beam diagnostics digital CCD camera LaserCam-HR II and BeamView version 4.8.1 software (Coherent). From this measurement, an effective diameter of 52 μm (86.5%) of the illumination beam was determined. The Gaussian fit (86.5%) coefficient of the spatial distribution of the light beam intensity was 0.988 (Figure 4b,c).

The *V*_PME_ voltage induced in the measuring coil was registered using an EG&G 5110 lock-in amplifier whose internal generator controlled the frequency of the laser beam chopping. In the field effect investigations, the back-gate bias was applied using a Keithley 2410 SourceMeter instrument.

The steady magnetic field induction was measured using an F.W. Bell 5080 tesla meter in the PME as well as in the Van der Pauw measurements. All measurements were performed at 294 K. The temperature was measured using a Lake Shore Cryotronics 211 temperature monitor. The experimental setups were computerized using an IEEE-488 bus and appropriate LabView programs. Figure 4d presents the main part of the measuring set up used for the PME investigations.

## Results and Discussion

**Figure 5:**
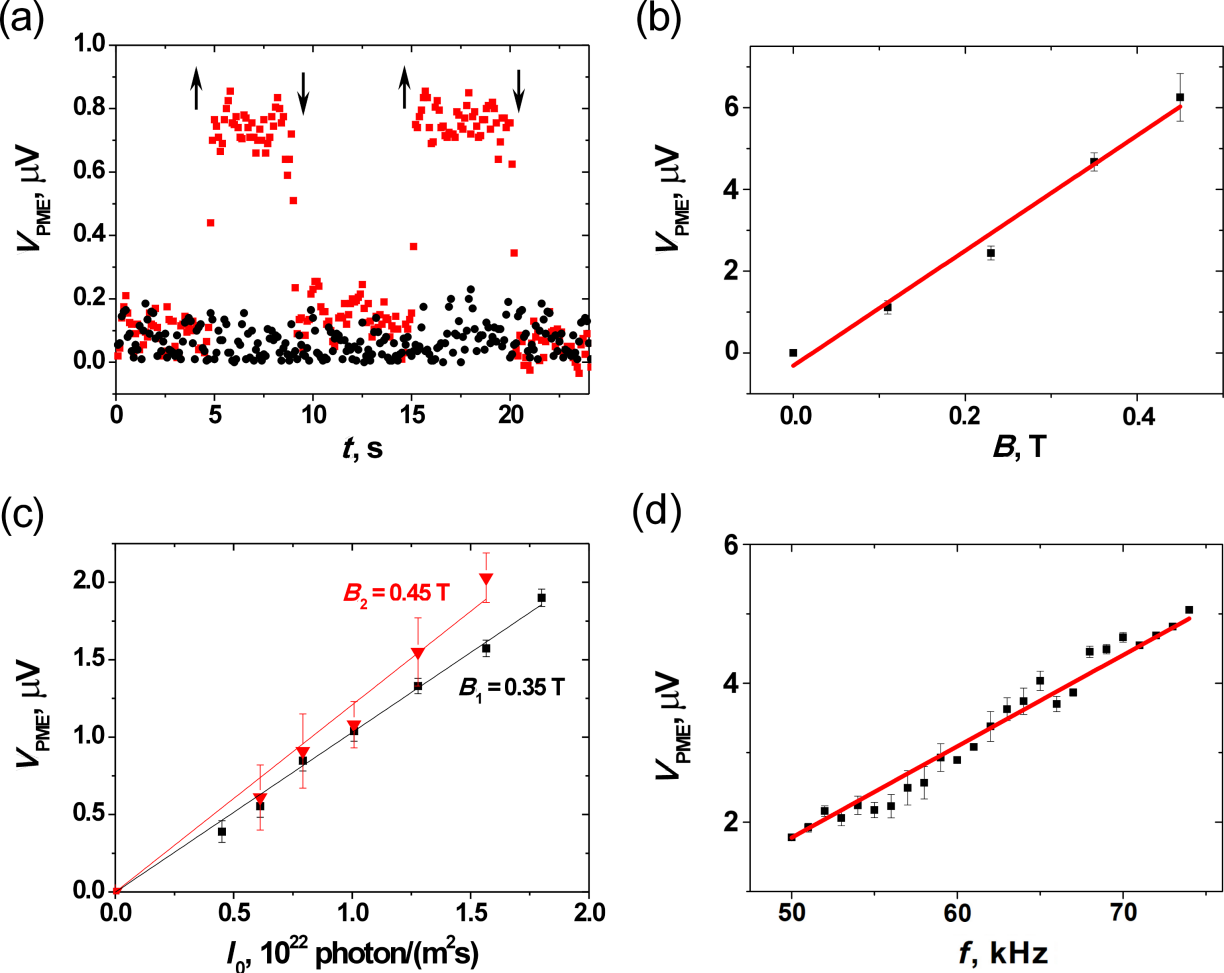
Voltage response in PME contactless investigations of graphene: (a) the time dependence for different values of magnetic field (black circles – *B* = 0; red squares – *B* = 0.45 T; *I* = 7.5·10^21^ photons/(m^2^s); *f* = 72 kHz; ↑ and ↓ – represent switch on and switch off points of sinusoidally modulated illumination); (b) dependence on magnetic field induction (*I* = 2.6·10^22^ photons/(m^2^s); *f* = 72 kHz); (c) dependence on illumination intensity for varying magnetic field (black squares – *B* = 0.35 T; red triangles – *B* = 0.45 T; *f* = 72 kHz ); (d) dependence on frequency of illumination chopping (*I* = 2.6·10^22^ photons/(m^2^s); *B* = 0.45 T); symbols – the experimental data; solid lines – linear dependences calculated for the best fitted values.

Figure 5a shows the induced *V*_PME_ response to the switching on and switching off of the sinusoidally modulated illumination of single-layer graphene with and without a magnetic field. The measured *V*_PME_ responses are proportional to the magnetic field induction (Figure 5b) and to the intensity of illumination (Figure 5c). Such dependences of *V*_PME_ are characteristic for the case of a carrier lifetime that is independent of the concentration of photogenerated carriers (i.e., for low illumination intensity) in a weak magnetic field [10]. These results agree with the first observations of the PME effect in graphene [27–28] and with the calculated dependence of the PME magnetic flux on the illumination intensity (Figure 2a). Simultaneously, the experimental results suggest small carrier mobility in the investigated non-suspended graphene. The last observation agrees with the carrier mobility μ_e_ = 1256(25) cm^2^V^−1^s^−1^ determined in the Van der Pauw measurements.

The linear dependence of *V*_PME_ on the frequency (*f*) of sinusoidal modulation of the illumination presented in Figure 5d is characteristic for the case 4π^2^*f*^2^τ^2^ << 1 (see Equation S19 in Supporting Information File 1) and suggests a short carrier lifetime.

**Figure 6:**
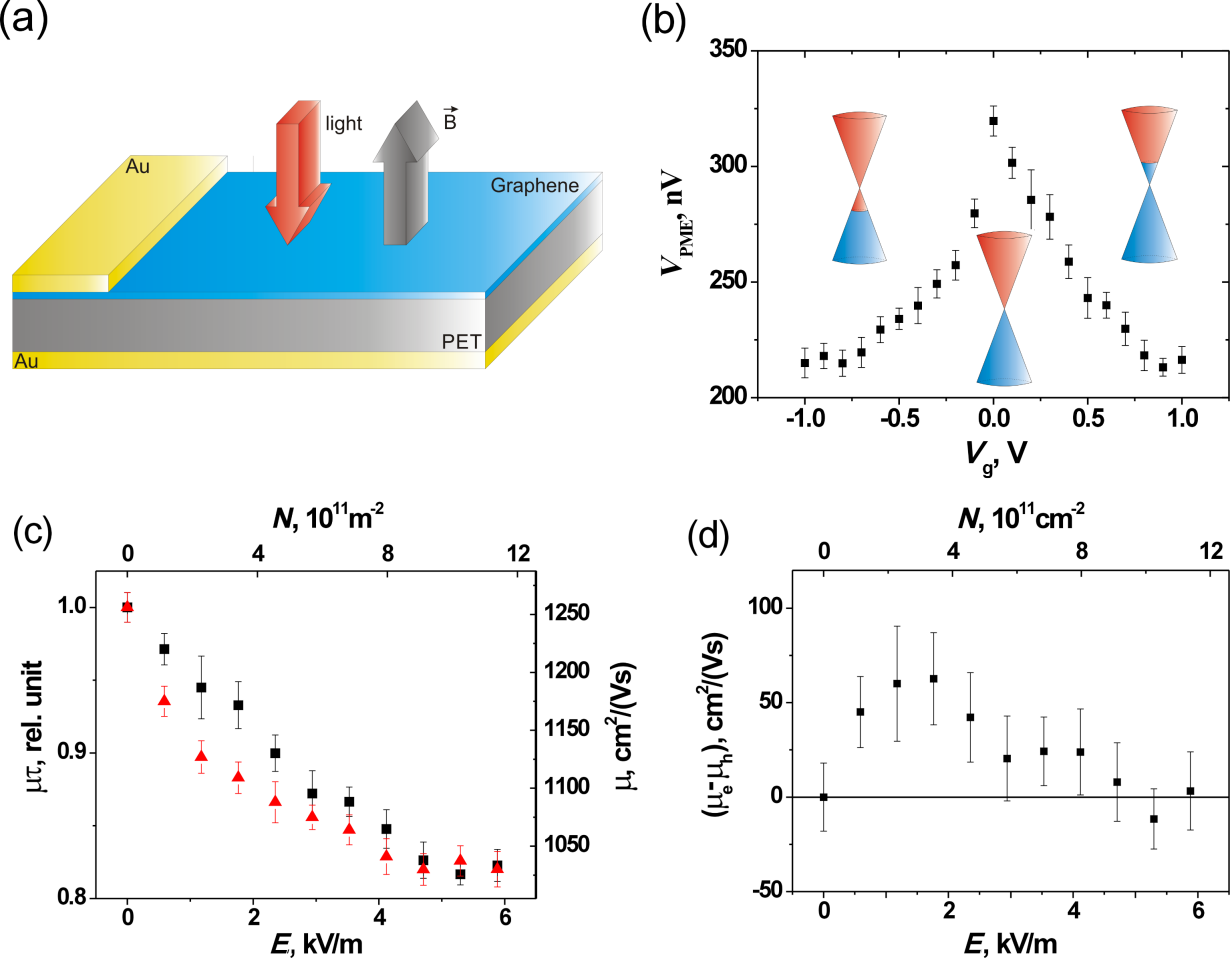
Schematic representation (a) of a back-gated sample used for the PME-Corbino investigations of graphene with electrostatically tunable carrier density; (b) PME voltage response vs gate voltage (*B* = 0.45 T; *I* = 2.6·10^22^ photons/(m^2^s); *f* = 72 kHz; the inserts depicts the positions of the Fermi energy in the conduction and valence bands); (c) product of mobility and lifetime (left axis) as well as the mobility (right axis) of electrons (black squares) and holes (red triangles) vs electric field (bottom axis) and concentration of electrostatically induced carriers (top axis); (d) difference between electron and hole mobilities vs electric field (bottom axis) and concentration of electrostatically induced carriers (top axis).

Unfortunately, the observed linear dependences of the PME response on experimental parameters (Figure 5b–d) have no forthright applicability for determining the parameters of investigated materials. Only a single value of one parameter can be determined from a linear fit to the experimental data with the theoretical dependence. Besides, it is rather difficult to deduce the above mentioned coefficient of proportionality between the measured *V*_PME_ voltage (induced in a measuring coil) and the investigated PME magnetic moment. However, the relative changes of a material parameter due to an external factor can be determined. Therefore, in this work, the PME-Corbino investigations of the dependence of carrier mobility on concentration of electrostatically tunable carrier density (*N*) were performed on a back-gated sample of graphene (Figure 6a) with known mobility and carrier concentration (μ_e_ = 1256(25) cm^2^V^−1^s^−1^ and *n*_e_ = 4.65(6)·10^16^ m^−2^ determined using Van der Pauw method).

It should be underlined that one of the most important properties of graphene [33–35] and other 2D materials [2,36–41] is the strong electric field effect which leads to electrostatically tunable carrier density. The charge carriers can change from electrons to holes with the application of an electrostatic gate. The switching takes place at the Dirac point, where the carriers have a minimum density. The gate voltage, *V*_g_, induces a sheet carrier concentration approximated by [33] (ε_0_ε/*we*)*V*_g_, where ε_0_ and ε are the permittivity of free space and the used dielectric, respectively; *e* is the electron charge; and *w* is the thickness of the dielectric. The authors of [35] provided the theoretical description of the current compact model of graphene field-effect transistors.

In our case, the energy position of the Fermi level of graphene was modulated by the field effect through the 170 μm thick PET foil. The relative permittivity of PET is ε = 3.5 at 71 kHz [42]. Figure 6b shows the PME voltage induced in the measuring coil as a function of the back-gate bias. The inserts depict the energy position of Fermi level and the switching between 2D electron and hole gases by changing the gate voltage. Taking into account the proportionality of the PME magnetic flux to the square of carrier mobility and lifetime presented above (Figure 2) (i.e., in the case of small values of μτ and weak magnetic field), one can find 
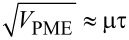
. Therefore, the left axis in Figure 6c presents the square root of the measured PME response (shown in Figure 6b) scaled as the μτ. The data are presented as a function of intensity of electric field (bottom axis) and concentration of electrostatically induced electrons and holes (top axis).

It was experimentally shown that the carrier lifetime only decreases slightly with carrier density in graphene [7]. Taking into account this approximation and the known value of the carrier mobility at the Dirac point (determined from Van der Pauw measurements), the data presented in Figure 6c can be normalized to values of electron and hole mobilities (see right axis in Figure 6c). It should be noted that in the case of different electron and hole mobilities, μ represents the so-called ambipolar carrier mobility [10].

The electron–hole asymmetry is concluded from the observed PME response in non-suspended graphene. It agrees with theoretical predictions [43] based on the influence of charged impurities scatterers on transport of carriers in graphene. Figure 6d shows the difference between electron and hole mobilities as a function of electric field (bottom axis) and concentration of electrostatically induced carriers (top axis). The interpretation of these results requires further theoretical analysis.

The presented PME method is restricted to photogenerated carriers and does not account for several groups of carriers with distinct mobilities, and in particular, hot electrons. A brief review [44] presented a number of experimental methods to determine carrier mobilities. These methods yield information on different mobilities (i.e., majority carrier mobility, or majority and minority carrier weighted “ambipolar” mobility). It should also be noted that carrier mobility values as determined by different methods of investigations can be drastically different. For instance, far-infrared magneto-transmission (FIR-MT) measurements show that graphene layers with very high carrier mobility can be observed in graphene films with low Hall effect mobility [45]. Further investigations should be performed on the same material using PME as well as other methods of determining carrier mobility.

## Conclusion

The investigation of complex phenomena in 2D materials requires the combination of multiple experimental techniques. The contactless PME method, used in Corbino geometry, is complementary to the mobility extraction methods based on field-effect measurements. In the case of small carrier mobility and weak magnetic field, the PME magnetic flux is proportional to the fourth power of the diffusion length of carriers. The contactless PME method can be used to obtain scan maps of local values of 2D semiconductor parameters, i.e., diffusion length, mobility or lifetime of carriers, in future investigations.

Possible difficulties in applying the proposed method may result from disturbances through the Corbino–Nernst effect from radial temperature gradients that are caused by heating of the center spot in the sample with the incident light beam. However, the distorting photovoltage and thermovoltage evoked due to illumination of the near-contact regions are eliminated in the proposed PME method. The existence of photogenerated hot carriers which can interact with the equilibrium free carriers must be taken into serious considerations in future experiments.

## Supporting Information

File 1 Theoretical description of the photomagnetoelectric effect in 2D materials in Corbino configuration.

## References

[1] Fiori G, Bonaccorso F, Iannaccone G, Palacios T, Neumaier D, Seabaugh A, Banerjee S K, Colombo L (2014). Nat Nanotechnol.

[2] Koppens F H L, Mueller T, Avouris P, Ferrari A C, Vitiello M S, Polini M (2014). Nat Nanotechnol.

[3] Yazyev O V, Kis A (2015). Mater Today.

[4] Bablich A, Kataria S, Lemme M C (2016). Electronics (Basel, Switz).

[5] Sul O, Kim K, Jung Y, Choi E, Lee S-B (2017). Nanotechnology.

[6] Sangwan V K, Hersam M C (2018). Annu Rev Phys Chem.

[7] Chen K, Yogeesh M N, Huang Y, Zhang S, He F, Meng X, Fang S, Sheehan N, Tao T H, Bank S R (2016). Carbon.

[8] Lin H, Braeuninger-Weimer P, Kamboj V S, Jessop D S, Degl’Innocenti R, Beere H E, Ritchie D A, Zeitler J A, Hofmann S (2017). Sci Rep.

[9] Zhong H, Zhang Z, Xu H, Qiu C, Peng L-M (2015). AIP Adv.

[10] Nowak M (1987). Prog Quantum Electron.

[11] Gassan-zade S G, Strikha M V, Shepelsky G A (2008). Semiconductors.

[12] Kostyuchenko V Y (2009). Optoelectron Instrum Data Process.

[13] Protasov D Y, Trifanov A V, Kostyuchenko V Y (2013). Eur Phys J: Appl Phys.

[14] Stariy S V, Sukach A V, Tetyorkin V V, Yukhymchuk V O, Stara T R (2017). Semicond Phys, Quantum Electron Optoelectron.

[15] Egorova S G, Chernichkin V I, Ryabova L I, Skipetrov E P, Yashina L V, Danilov S N, Ganichev S D, Khokhlov D R (2015). Sci Rep.

[16] Galeeva A V, Egorova S G, Chernichkin V I, Tamm M E, Yashina L V, Rumyantsev V V, Morozov S V, Plank H, Danilov S N, Ryabova L I (2016). Semicond Sci Technol.

[17] Galeeva A V, Krylov I V, Drozdov K A, Knjazev A F, Kochura A V, Kuzmenko A P, Zakhvalinskii V S, Danilov S N, Ryabova L I, Khokhlov D R (2017). Beilstein J Nanotechnol.

[18] Hazrati R, Shojaei S, Karimi M, Kalafi M (2010). Infrared Phys Technol.

[19] Oskolkov B, Filonov O, Prussak N (2016). Proc SPIE.

[20] (2018). IR Detectors. Room Temperature and TE-Cooled.

[21] Mette H (1966). Corbino-PME effect as a possible tool microelectronic materials evaluation: theory, Technical Report ECOM 2652.

[22] Hlávka J (1983). Rev Sci Instrum.

[23] Hlávka J (1981). Rev Sci Instrum.

[24] Loncierz B, Muni R, Nowak M (1995). Thin Solid Films.

[25] Loncierz B, Nowak M (1997). Proc SPIE.

[26] Nowak M, Solecka B (2000). Vacuum.

[27] Nowak M, Solecka B, Jesionek M (2014). Acta Phys Pol, A.

[28] Nowak M, Solecka B, Jesionek M (2015). MRS Online Proc Libr.

[29] Peters E C, Giesbers A J M, Burghard M, Kern K (2014). Appl Phys Lett.

[30] Schmidt B A, Bennaceur K, Bilodeau S, Gervais G, Pfeiffer L N, West K W (2015). Solid State Commun.

[31] Kumar M, Laitinen A, Hakonen P (2018). Nat Commun.

[32] Bonaccorso F, Sun Z, Hasan T, Ferrari A C (2010). Nat Photonics.

[33] Novoselov K S, Geim A K, Morozov S V, Jiang D, Zhang Y, Dubonos S V, Grigorieva I V, Firsov A A (2004). Science.

[34] Meric I, Han M Y, Young A F, Ozyilmaz B, Kim P, Shepard K L (2008). Nat Nanotechnol.

[35] Lu N, Wang L, Li L, Liu M (2017). Chin Phys B.

[36] Wang Q H, Kalantar-Zadeh K, Kis A, Coleman J N, Strano M S (2012). Nat Nanotechnol.

[37] Perera M M, Lin M-W, Chuang H-J, Chamlagain B P, Wang C, Tan X, Cheng M M-C, Tománek D, Zhou Z (2013). ACS Nano.

[38] Jo S, Costanzo D, Berger H, Morpurgo A F (2015). Nano Lett.

[39] Das S (2016). Sci Rep.

[40] Sung J H, Heo H, Si S, Kim Y H, Noh H R, Song K, Kim J, Lee C-S, Seo S-Y, Kim D-H (2017). Nat Nanotechnol.

[41] Giannazzo F, Greco G, Roccaforte F, Sonde S S (2018). Crystals.

[42] Yang P, Tian F, Ohki Y (2014). IEEE Trans Dielectr Electr Insul.

[43] Stauber T, Peres N M R, Castro Neto A H (2008). Phys Rev B.

[44] Karl N (2003). Synth Met.

[45] Jernigan G G, VanMil B L, Tedesco J L, Tischler J G, Glaser E R, Davidson A, Campbell P M, Gaskill D K (2009). Nano Lett.

