# Supplementary material for: Contactless photomagnetoelectric investigations of 2D semiconductors
Source: Beilstein J Nanotechnol. 2018 Oct 25;9:2741–9. doi: 10.3762/bjnano.9.256 (PMC6204781; doi:10.3762/bjnano.9.256)
Supplement: File 1 — Theoretical description of the photomagnetoelectric effect in 2D materials in Corbino configuration. [file Beilstein_J_Nanotechnol-09-2741-s001.pdf]

Supporting Information

for

Contactless photomagnetoelectric investigations of 2D semiconductors

Marian Nowak[1]*, Marcin Jesionek[1], Barbara Solecka[1], Piotr Szperlich[1], Piotr Duka[1] and Anna Starczewska[1]

Address: [1]Institute of Physics, Center for Science and Education, Silesian University of Technology, Krasińskiego 8, 40-019 Katowice, Poland

* Corresponding author

Email: Marian Nowak - marian.nowak@polsl.pl

Theoretical description of the photomagnetoelectric effect in 2D materials in Corbino configuration

The transport of electrons and holes through 2D samples in the presence of an electric field ($\vec{\boldsymbol{E}}$) and steady magnetic field ($\vec{\boldsymbol{B}}$), under photogeneration and recombination is described by the following equations [S1]

$$\vec{J_e} = e\mu_e n_e \vec{E} + eD_e grad(n_e) - \mu_{He}(\vec{J_e} \times \vec{B}) \quad \text{(S1)}$$

$$\vec{J_h} = e\mu_h n_h \vec{E} - eD_h grad(n_h) + \mu_{Hh}(\vec{J_h} \times \vec{B}) \quad \text{(S2)}$$

$$\frac{\partial n_e}{\partial t} = G_e - R_e + \frac{1}{e} div(\vec{J_e}) \quad \text{(S3)}$$

$$\frac{\partial n_h}{\partial t} = G_h - R_h - \frac{1}{e} div(\vec{J_h}) \quad \text{(S4)}$$

where $\vec{J_e}$ and $\vec{J_h}$ are vectors for the electron and hole current densities, that can be written in the radial and azimuthal components J_{re}, J_{ae} , J_{rh} and J_{ah}

$$\vec{J_e} = J_{re}\vec{e}_r + J_{ae}\vec{e}_a \quad \text{(S5)}$$

$$\vec{J_h} = J_{rh}\vec{e}_r + J_{ah}\vec{e}_a \quad \text{(S6)}$$

e is absolute value of electric charge, μ_e, μ_h, μ_{He} and μ_{Hh} are drift and Hall mobilities for electron and holes, D_e and D_h are the diffusion constants for the electrons and holes, n_e and n_h are concentrations of equilibrium for the electrons and holes, G_e, G_h, R_e and R_h are photogeneration and recombination rates for the electrons and holes, t represents time, $\overrightarrow{e_r}$ and $\overrightarrow{e_a}$ are versors of the radial and azimuthal axis.

The electric field is determined by the gradient of the potential (V_E):

$$\vec{E} = -grad(V_E). \qquad (S7)$$

Then, by Gauss's law, the potential satisfies Poisson's equation that can be written for 2D sample in the form [S2]:

$$div[\varepsilon_r \varepsilon_0 grad(V_E)] = \rho_{free}. \qquad (S8)$$

where ρ_{free} is the free charge density, ε_r is the relative dielectric permittivity of the medium and ε_0 is the vacuum permittivity.

In the case of a small and steady illumination with interband (intrinsic) photoexcitation of the electrons and holes, the following simplification can be applied

$$R_e = R_h = \frac{\Delta n_e}{\tau} \qquad (S9)$$

and

$$G_e = G_h = \alpha\, I_{\mathrm{v}}(r) \qquad (S10)$$

where Δn_e represents the concentration of excess electrons, τ is the effective carrier lifetime, α is the absorption coefficient of light (i.e. α = 1/137 for graphene [S3]), r is the distance from the center of light spot, and $I_v(r)$ represents the intensity (in photons) of light incident upon the sample. For example, in the case of illumination of a sample with a TEM_{00} laser beam

$$I_{\mathrm{v}}(\mathrm{r}) = I_{\mathrm{v}0}\, exp\left[-2\left(\frac{r}{R_b}\right)^2\right] \qquad (S11)$$

where R_b represents beam radius at $I_{\mathrm{v}}(R_b) = I_{\mathrm{v}0}\, e^{-2}$, I_{v0} is the maximum intensity of the radiation.

When considering $\frac{\partial n_e}{\partial a} = 0$ and $\frac{\partial n_h}{\partial a} = 0$ **,** for the case of μ_e=μ_h=μ_{He}=μ_{Hh}=μ, the solution of Eqns (1) to (4) becomes represented by the following equation

$$\frac{\partial^2 \Delta n_e}{\partial r^2} + \frac{1}{r}\frac{\partial \Delta n_e}{\partial r} + \frac{1+\mu^2 B^2}{D\tau}\,\Delta n_e = \frac{1+\mu^2 B^2}{D}\,\alpha I_{\mathrm{v}}(r) \tag{S12}$$

with boundary conditions:

$$\lim_{r\to\infty} \Delta n_e = 0 \tag{S13}$$

$$\lim_{r\to\infty} \frac{\partial \Delta n_e}{\partial r} = 0 \tag{S14}$$

It should be noted that in the case of different mobilities of electrons and holes, μ represents so-called ambipolar mobility of carriers; it is known that the PME effect is determined by the so-called effective ambipolar carrier lifetime, ambipolar carrier diffusion constant, and ambipolar diffusion length of carriers [S1].

Equation (10) can be solved numerically. Using this solution, the density of total Corbino-PME current can be given by

$$\mathrm{J_a(r)} = \mathrm{J_{ae}} + \mathrm{J_{ah}} = 2\,\mu\,\mathrm{B}\,\frac{\mathrm{eD}}{1+\mu^2\mathrm{B}^2}\frac{\partial \Delta \mathrm{n_e}}{\partial \mathrm{r}} \tag{S15}$$

The integral magnetic moment of the PME current distribution is represented by

$$\overrightarrow{M_b} = \int_0^\infty 2\,\pi\,(\vec{r}\,\times \vec{e}_a)\,J_a(r)\,dr \tag{S16}$$

Mechanical torque acting on a sample with an integral magnetic moment of a PME current distribution in an external magnetic field is given by

$$\vec{N} = \overrightarrow{M_b} \times \vec{B} \tag{S17}$$

and the magnetic flux density $\overrightarrow{B_{PME}}$, evoked by the PME circulating current, is given by [S4]

$$\vec{B}_{PME}(\vec{r}) = \frac{\mu_0}{4\pi}\left[\frac{3\vec{r}\left(\overrightarrow{M_b}\cdot\vec{r}\right)}{r^5} - \frac{\overrightarrow{M_b}}{r^3}\right] + \frac{2\mu_0}{3}\,\overrightarrow{M_b}\,\delta^3(\vec{r}) \tag{S18}$$

where μ_0 is magnetic permeability of the free space, and δ^3 is the three-dimensional Dirac delta function.

For sinusoidal modulation of the illumination with frequency *f*, the following is true

$$I_{Vt}(r,t) = I_V(r)\left[\frac{1}{2} - \frac{1}{2}cos\left(2\,\pi\,f\,t\right)\right] \tag{S19}$$

thus, according to [S5], one obtains the following

$$J(r,t) = A\,\frac{I_{Vt}(r,t)}{\sqrt{1+4\pi^2 f^2 \tau^2}} \tag{S20}$$

where A represents the independent time function of the sample and the experiment parameters (e.g. carrier lifetime, carrier mobilities, external magnetic field). The total PME magnetic flux is equal to the integral of the magnetic fluxes created by the elementary Corbino-PME currents J(r,t)dr.

According to the Faraday law, the voltage V_{PME} induced in the measurement coil during contactless PME investigations is proportional to the derivative of PME magnetic flux versus time. Because the magnetic flux is proportional to the intensity of PME current, then

$$V_{PME} = A_0\,\frac{\partial\overrightarrow{M_b}}{\partial t} = A_1\,\frac{f}{\sqrt{1+4\pi^2 f^2 \tau^2}} \tag{S21}$$

where A_0 and A_1 represent coefficients independent of frequency. These coefficients represent not only the sample, but also the apparatus constant, which is difficult to determine for absolute contactless investigations.

References

[S1] Nowak M., Solecka B., Jesionek M., Determination of diffusion length of carriers in graphene using contactless photoelectromagnetic method of investigations, *MRS Proc.* **1727** mrsf14-1727-k06-06 (2015) http://dx.doi.org/10.1557/opl.2015.68

[S2] Feijoo P.C., Jiménez D., Cartoixà X., Short channel effects in graphene-based field effect transistors targeting radio-frequency applications, 2D Mater. 3 (2016) 025036 (2016) http://dx.doi.org/10.1088/2053-1583/3/2/025036

[S3] Bonaccorso F., Sun Z., Hasan T., Ferrari A. C., Graphene photonics and optoelectronics, *Nat. Photon.* **4** 611-622 (2010) http://dx.doi.org/10.1038/NPHOTON.2010.186

[S4] Torrez E., Components, Laws and concepts of electromagnetism, Library Press, New York (2016) 101-103.

[S5] Choo S.C., Etchells A.M., Watt L.A.K., High-frequency photoelectromagnetic effect in p-type InSb, *Phys. Rev. B* **4** 4499-4511 (1971) http://dx.doi.org/10.1103/PhysRevB.4.4499
